# Assessing the influence of COVID-19 lockdown measures on cognition and behavior in school age children in Arba Minch Health and Demographic Surveillance site, Southern Ethiopia: A cross-sectional study

**DOI:** 10.1371/journal.pgph.0002978

**Published:** 2024-03-21

**Authors:** Befikadu Tariku Gutema, Eshetu Zerihun Tariku, Zeleke Aschalew Melketsedik, Bruno Levecke, Stefaan De Henauw, Amina Abubakar, Souheila Abbeddou

**Affiliations:** 1 School of Public Health, College of Medicine and Health Sciences, Arba Minch University, Arba Minch, Ethiopia; 2 Department of Public Health and Primary Care, Faculty of Medicine and Health Sciences, Ghent University, Ghent, Belgium; 3 School of Nursing, College of Medicine and Health Sciences, Arba Minch University, Arba Minch, Ethiopia; 4 Department of Translational Physiology, Infectiology and Public Health, Faculty of Veterinary Medicine, Ghent University, Merelbeke, Belgium; 5 Kenya Medical Research Institute (KMRI)–Wellcome Trust Research Programme, Centre for Geographic Medicine Research (Coast), Kenya Medical Research Institute, Kilifi, Kenya; Politecnico di Milano, ITALY

## Abstract

Ethiopian schools were closed for nearly 40 weeks as a measure to control the COVID-19 pandemic. The objective of the study was to evaluate the role of COVID-19 pandemic’s restrictive measures on cognition and behavioral difficulties of schoolchildren in Arba Minch Health and Demographic Surveillance Site. Two cross-sectional surveys were conducted in November 2019 (pre-COVID-19-lockdown) and November 2020 (post-COVID-19 lockdown). Data were collected both at the school and homes of the children. Cognitive development of the children was assessed using digit span, Raven’s coloured progressive matrices (RCPM) and Visual search using cancellation task. Behavioral difficulties score of the children was assessed using Strengths and Difficulties Questionnaire. Analysis of covariance (ANCOVA) was used to compare between the cognition outcomes and behavioral difficulties score pre- and post-COVID-19-lockdown. In a sub-group of children who provided data in both surveys, the difference in cognitive and behavioral outcomes was tested using a mixed effect model. Compared to the pre-COVID-19-lockdown, schoolchildren post-COVID-19-lockdown scored lower in the standardized performance index for the visual search task, which measures attention (0.27 SD, 95% confidence intervals (95%CI): −0.40, −0.13). However, they scored higher by 0.26 SD (95%CI: 0.13, 0.40) and 0.36 SD (95%CI: 0.22, 0.49) in digit span and RCPM, respectively, measuring working memory and non-verbal intelligence. There was no significant difference in total difficulties score between pre- and post-COVID-19-lockdown (0.01 SD, 95%CI: -0.12, 0.15). The subgroup analysis showed a significant increase in digit span among children post-COVID-19-lockdown while the other domains did not show a significant change. Factors contributing to the improvement of children’s cognitive domains while away from the school environment should be explored and utilized to enhance child development. This study was registered at clinicaltrials.gov as NCT04137354 on October 14, 2019.

## Introduction

Severe acute respiratory syndrome coronavirus 2 (SARS-CoV-2) that causes coronavirus disease (COVID-19) is a serious disease which affected the entire world in an unprecedented way [1]. Because of the high contagious nature of the virus, different restrictive measures have been taken to control and break the exponential infection curve of COVID-19, from total lockdown to only some restrictions on gathering and physical distancing [2]. School closure was one of the widely implemented physical distancing measures while maintaining remote learning when possible [3]. This measure was implemented despite the very low fatality risk of the COVID-19 pandemic on school age children compared to the older population. Infection fatality risk for confirmed COVID-19 was less than 1 per 10,000 in school age children, based on nationwide sero-epidemiological study in Spain [4]. At the end of March 2020, 169 countries had closed their schools which affected nearly 1.5 billion students [5]. During the closure of the schools, countries started different types of mitigation strategies for engaging the students in remote learning activities. These included online platforms, television, radio, and paper-based take-home packages [6]. These forms of education systems are not equally available to all throughout the countries. Most of the methods require electricity, computer (maybe smartphone), internet, applications, radio, television, printing materials and/or capacity to use them (including support from parents) [3]. The online platforms also showed differences in their outcomes based on the child’s mental and behavioral status [7].

The Ethiopian government started its restrictive measures on March 13, 2020 [8]. The school closure lasted for nearly 40 weeks, which affected around 20 million primary schoolchildren in the country [5, 8, 9]. The Ethiopians used paper-based take-home course packages, and broadcasted learning programs through radio and television [10]. However, these measures were unequally distributed and, consequently, rural children were at a disadvantage. Based on the Ethiopian Demographic and Health Survey of 2016, only 28.2% and 13.8% households possess radio and television, respectively, with significantly lower possession of television among rural residents (only 2.1%) [10, 11]. Additionally, these children are usually from households whose parents have no formal education, limiting their support to their children.

Schooling is a strong instrument to promote development, mental health and growth of children. In low-income countries, school is an opportunity for development and academic enrichment, especially for girls, children from disadvantaged socioeconomic groups, and those whose family environment lacks any formal education [12–15]. For most of the children whose parents are illiterate, or do not have time and resources for supporting their kids, school is the only place where they get the basis for their development [16]. Studies showed that socioeconomically disadvantaged children gain less academic skills during school breaks such as summer vacation [13]. Contrarily, children from socioeconomically disadvantaged households with good school attendance have a great chance to close the gap with those with higher socioeconomic status [15, 16]. In addition, schools and communities constitute an important environment for promoting mental health among children [17]. Reduced social interactions during the restrictive measures affected negatively their mental health [18].

When the first restrictive measures started in Ethiopia, a study assessing the effects of intermittent iron supplementation and semestrial high-dose vitamin A on schoolchildren (7–10 years) in South Ethiopia was ongoing. The study was interrupted and was reconducted when schools resumed their activities in October 2020. During both rounds, data on cognitive development, behavioral difficulties and Home Observation for Measurement of the Environment (HOME) scale were collected. The objective of this study is to evaluate the role of COVID-19 pandemic’s restrictive measures on cognition and behavioral difficulties of schoolchildren in Arba Minch Health and Demographic Surveillance Site (HDSS), using institutional-based cross-sectional study design for analyzing the data collected in November 2019 (pre-COVID-19-lockdown) and November 2020 (post-COVID-19-lockdown) in two different cohorts.

## Materials and methods

### Study area

The study was conducted in HDSS, which covers communities of Arba Minch Zuria and Gach Baba districts in Gamo zone, Southern Ethiopia. Both districts have 31 kebeles (the smallest administrative unit in Ethiopia), representing 71 public primary schools. The study site included 9 kebeles and 22 public primary schools.

#### Study design, period and population

In November 2019, we collected baseline data of the interventional randomized clinical trial titled “The effects of intermittent iron and vitamin A supplementation on nutritional status and development of schoolchildren in Arba Minch HDSS, Southern Ethiopia”. In March 2020, the government of Ethiopia implemented a suite of restrictive measures, including the closure of schools. Consequently, the intervention and the data collection were terminated. In November 2020, the study was reconducted with newly recruited study subjects. The two cross-sectional surveys included 7–10 year aged children who attended one of the nine randomly selected public primary schools (one school per kebele).

#### Sample size determination

The sample size was calculated for the randomized controlled trial to detect an effect size of 0.3 standard deviation (SD) in child anemia and cognitive development scores with 5% level of significance and 80% power. Considering a 2x2 factorial design and a 30% dropout rate, 252 children per group are required, totaling 504 schoolchildren.

#### Sampling procedures

Nine kebeles were included in this study, and to ensure the harmonization of school selection, one public primary school near the center of the HDSS kebele was included. Immediately after the end of registration (at the beginning of the academic year), rosters of grades 1 to 4 were collected from each school. These rosters included the name of the enrolled pupil and their age. Those children who were below 7 and above 10 years of age were excluded from the rosters, and sequential numbers were given for each school. Using Microsoft Excel, numbers were randomly selected and the corresponding child was screened and included in the study if they were eligible.

#### Inclusion and exclusion criteria

To be eligible, schoolchildren needed: (1) to be aged 7–10 years, (2) parental informed consent, with agreement from the child to partake in the study, (3) parents intending to remain in the kebele throughout the entire academic year, and (4) acceptance of the intervention package, which involved blood draw and home visits. Exclusion criteria included children with (1) chronic conditions like diabetes and asthma, or (2) night blindness (a type of vitamin A deficiency) or any severe form of vitamin A deficiency.

#### Data collection procedure

Data that are presented in this study were collected both at the school and the home of the children by four different teams. Children were enrolled at the school by the enrolment team (BSc. in Nursing/Health Officers). Cognitive development assessors (another team with BSc. in Nursing/Health Officers) collected the data on paper, which was entered afterward on Research Electronic Data Capture (RedCap). Two teams of HDSS data collectors visited the child’s home to collect Home Observation for Measurement of the Environment (HOME) scale and Strengths and Difficulties Questionnaire (SDQ), and child and household characteristics. All data collection materials were originally developed in English and translated to and administered in Amharic. Except for cognitive data, all data were collected digitally and managed using REDCap electronic data capture tools hosted at the Ghent University [19, 20], offline using the REDCap Mobile App [21], which were collected on paper, and then entered on the REDCap database.

Cognitive development of the children was assessed using digit span (forward and backward), visual search using cancellation task, and Raven’s coloured progressive matrices (RCPM) test. In the forward digit span, the child is instructed to repeat the numbers spoken by the examiner. In the backward digit span, the child repeats the numbers in the reverse order of those spoken. The examiner utters the numbers with a consistent pitch and a rate (1 number per second). The highest digit to which the child responded correctly was recorded. The RCPM instrument contains three sections (A, Ab, and B), each comprising 12 items. In each item, subjects are presented with an incomplete design and six alternatives, from which they must choose the one that best completes the design. Each correctly solved item results in a score 1. Visual search using the cancellation task consists of a pattern of symbols interspersed with target symbols, with the instruction to cross out (cancel) all the targets. The task involves visual search for the identification of the target and either cancelling or circling the target. The time taken to finish the task and the number of correctly crossed-out (canceled) targets were recorded.

At the children’s homes, SDQ was collected from parents to assess the behavioural difficulties of the children. SDQ includes emotional symptoms, conduct problem, hyperactivity, peer problem and prosocial behaviour [22]. The same team also collected the HOME score, which was used in the assessment of stimulation around the home environment [23]. In addition, questionnaires were used to collect socio-demographic characteristics of the child, their caregiver and their household at the children’s homes. Information on food security using household food insecurity access scale [24, 25], household characteristics and ownership of assets, and history of illness during the previous two weeks were collected from the children’s caregivers by another team.

#### Data quality control

We included different data collection teams for assessing different aspects of the child’s, caregiver’s and household’s characteristics. Proper training was given for all the data collection teams, and a refresher training was organized before the start of the post-COVID data collection. We selected, trained, and implemented cognitive development assessment tools with expert support (AA).

### Ethical approval and consent to participate

The study was conducted in accordance with the Declaration of Helsinki. Ethical clearance was obtained from the National Research Ethics Review Committee of Ministry of Science and Higher Education of Ethiopia (P.S.M/14.1/505/20), and the Commission on Medical Ethics of Ghent University Hospital (EC/2019/1289). Formal letters were submitted to the concerned bodies to get permission (district administration, district health and education office, kebele’s office and schools). School directors and school teachers participated in sensitization meetings. Written Informed consent was obtained from the guardian of the child. The procedure was explained to the child, whose agreement was a condition for enrollment. The study has been registered at clinicaltrials.gov (reference number NCT04137354). All children were treated with albendazole (Aldaz 400 mg tablet, Bayberry Pharmaceuticals Pvt Ltd) for intestinal parasites following the guideline of the Ministry of Health of Ethiopia [26]. Children diagnosed with schistosomiasis and *Taenia saginata*, were treated with praziquantel (40 mg/kg praziquantel 600 mg, Leben Laboratories, India, Pvt. Ltd.).

### Statistical analysis

Statistical analysis was done using Stata 14 statistical software. The dependent variables are cognitive development of schoolchildren collected using digit span, RCPM test and visual search using cancellation task, and total difficulties score collected using SDQ. The total digit span is the sum of the two scores of digit span forward and backward. The RCPM score is the total number of correct responses with the minimum of zero and the maximum of 36. Performance index was estimated based on the Geldmacher 1998 using the visual search using cancellation task by multiplying correct response per total target and correct response per total time to complete the task [27]. Total difficulties score is the sum of scores given to emotional symptoms, conduct problems, hyperactivity, and peer problems. The outcomes of cognitive development were standardized to a mean of 0 and a standard deviation of 1 (Z-score) by their class grade. The normality of continuous variables was checked (Shapiro–Wilk test >0.05) and all the dependent outcomes were transformed using a two-step approach [28]. Borderline and abnormal difficult behaviors were determined using the cutoff points indicated by Goodman [22]. HOME score items were normalized to make their contribution for generation of index equal and summed. A home environment was considered less stimulating if the HOME score was lower than 25 percentiles. The wealth index was constructed using the household characteristics, assets and availability of farm animals using principal component analysis (PCA) [29]. Household Food Insecurity Access Prevalence was used to categorize households into food insecure access by merging mild, moderately and severely food insecure and food secure otherwise [25].

Unpaired t-test for continuous variables and Chi-square test for categorical variables were used to assess the difference between the characteristics of the participants during pre- and post-COVID-19 restrictive measures. Analysis of covariance (ANCOVA) was used to compare between dependent outcomes before and after COVID-19 restrictive measures after checking normality of residuals. The differences in outcomes were significant at a *p*-value of less than 0.05. In a bivariate analysis, associations between the dependent variables and a list of independent variables including child’s sex and age, caretaker and head of the household education, HOME score, and difficulties at home were tested. Variables with p-value less than 0.20 were included as covariates in the ANCOVA models for adjustment. The modifying effect of total difficulties and prosocial behavior (borderline and abnormal together), and home stimulating environment on the cognitive development domains were assessed. Variables examined as potential modifiers were considered only when the interaction between the modifying variable and enrollment year with the outcomes resulted in a *p*-value <0.10. In a subgroup analysis among school children who participated in both surveys, we assessed the change in cognitive and behavioral outcomes using linear mixed effect model with the child as a random effect, including the aforementioned covariates.

Collinearity was checked for independent variables by estimating variable inflation factor (VIF). Outliers were checked by predicting Studentized residual, their effect were assessed, and appropriate measures were taken based on the result. Homoscedasticity of residuals were checked using Breusch-Pagan/Cook-Weisberg test. Effect sizes (partial eta squared (η^2^_p_)) were used to describe between-group differences.

## Results

### Characteristics of the participating children, their caregivers and households

Data were collected from 994 children, and those who participated in both surveys were exclusively included in the pre-COVID-19-lockdown dataset. In total, 487 and 419 schoolchildren participated in the two cross-sectional surveys, during pre- and post -COVID-19-lockdown, respectively. Eighty-eight children participated in both pre-and post-COVID-19 lockdown surveys. The mean (SD) age of the children was 9.65 years (1.80) and 9.53 years (1.58), in the first and the second survey, respectively, with no significant difference. More children were recruited from lower grade during post-COVID-19-lockdown (p <0.01). There were more children with recent illnesses pre-COVID-19-lockdown. There were more caretakers with formal education, and more households which food secure post-COVID-19-lockdown. The average HOME score was higher among participants during pre-COVID-19-lockdown survey, while the average wealth index of the household were higher post-COVID-19-lockdown (**Table 1**).

**Table 1:** Socio-demographic characteristics of the schoolchildren and their household by survey year (n = 906).

Variable	Categories	Pre-	Post––	Total	χ-square /t-test	p-value
Age (Mean (SD))		9.65 (1.80)	9.53 (1.58)	9.59 (1.70)	1.00^∝^	0.317
Child grade (class) (n (%))	1	133 (27.3)	134 (32.0)	267 (29.5)	27.84	<0.01
	2	134 (27.5)	165 (39.4)	299 (33.0)		
	3	138 (28.3)	78 (18.6)	216 (23.8)		
	4	82 (16.8)	42 (10.0)	124 (13.7)		
Sex (n (%))	Male	261 (53.6)	217 (51.8)	478 (52.8)	0.29	0.588
	Female	226 (46.4)	202 (48.2)	428 (47.2)		
Recent Illness (n (%))	Yes	137 (28.1)	55 (13.1)	192 (21.2)	30.36	<0.01
	No	350 (71.9)	364 (86.9)	714 (78.8)		
Caretaker’s educational status (n (%))	Illiterate	273 (56.1)	208 (49.6)	481 (53.1)	6.55	0.038
	1–8	180 (37.0)	164 (39.1)	344 (38.0)		
	9 & above	34 (7.0)	47 (11.2)	81 (8.9)		
Household head’s educational status (n (%))	Illiterate	241 (49.5)	196 (46.8)	437 (48.2)	3.17	0.205
	1–8	188 (38.6)	156 (37.2)	344 (38.0)		
	9 & above	58 (11.9)	67 (16.0)	125 (13.8)		
Wealth score (Mean (SD))		-0.46 (2.45)	0.33 (2.49)	-0.09 (2.51)	-4.75^∝^	<0.01
Food security status (n (%))	Insecure	193 (39.6)	215 (51.3)	408 (45.0)	12.42	<0.01
	Secure	294 (60.4)	204 (48.7)	498 (55.0)		
HOME Score (Mean (SD))		13.41 (3.92)	12.70 (4.31)	13.08 (4.12)	2.60^∝^	<0.01

Pre-: Pre-COVID-19-lockdown; Post-: Post-COVID-19-lockdown; ^∝^Unpaired *t*-test

### Behavioral and cognitive characteristics of the participating children

The mean (SD) during pre- and post-COVID-19-lockdown were, respectively, for behavioral difficulties score 8.78 (5.47) and 9.50 (5.78) (p = 0.054), and for prosocial behavior score 7.71 (2.17) and 7.42 (2.24) (p = 0.05).

Around 11% and 14% of the children had behavioral difficulties during pre- and post-COVID-19-lockdown, respectively. Abnormal prosocial behaviors were diagnosed in 9.2% and 13.6% of the children during pre- and post-COVID-19-lockdown, respectively. The highest prevalence of behavioral problem was found with children having peer problems; 20.7% and 23.9% during pre- and post-COVID-19-lockdown, respectively. The proportions of children with borderline and abnormal difficulties and prosocial behaviors score were no significantly different between the two surveys (**Table 2**). The total difficulties score was not significantly different between participating children, pre- and post-COVID-19-lockdown (F (1,899) = 0.04, p = 0.847, η^2^_p_ = 4.20E-05) (**Table 3**).

**Table 2:** Cut-offs proportions of the SDQ scales and comparison between pre- and post-COVID-19-restrictions of schoolchildren at Arba Minch HDSS, Southern Ethiopia (n = 906).

Behavioral difficulties score	Normal (N (%))		Borderline (N (%))		Abnormal (N (%))		χ² test	p value
	Pre-	Post-	Pre-	Post-	Pre-	Post-		
Total Difficulty	391 (80.3)	319 (76.1)	44 (9.0)	40 (9.6)	52 (10.7)	60 (14.3)	2.98	0.226
Hyperactivity	445 (91.4)	374 (89.3)	28 (5.8)	29 (6.9)	14 (2.9)	16 (3.8)	1.21	0.546
Emotional Symptoms	369 (75.8)	320 (76.6)	41 (8.4)	39 (9.3)	77 (15.8)	60 (14.3)	0.54	0.762
Conduct Problems	336 (69.0)	278 (66.4)	50 (10.3)	52 (12.4)	101 (20.7)	89 (21.2)	1.18	0.555
Peer Problems	295 (60.6)	233 (55.6)	91 (18.7)	86 (20.5)	101 (20.7)	100 (23.9)	2.34	0.311
Prosocial Behavior	390 (80.1)	328 (78.3)	52 (10.7)	34 (8.1)	45 (9.2)	57 (13.6)	5.46	0.065

Pre-: Pre-COVID-19-lockdown; Post-: Post-COVID-19-lockdown

**Table 3:** Standardized means and ANCOVA result of cognitive developmental and behavioral difficulty of schoolchildren at Arba Minch HDSS before and after lockdown of the school due to COVID 19 pandemic (n = 906).

Cognitive outcome	COVID-19 lockdown	Mean (95% CI)		F (df)	p-value	η^2^p
		Unadjusted	Adjusted*			
Digit span^1^	Before	-0.27 (-0.36, -0.18)	-0.26 (-0.35, -0.17)	14.21 (1,902)	<0.01	0.016
	After	0.01 (-0.09, 0.11)	-0.002 (-0.10, 0.10)			
	Difference	0.28 (0.14, 0.41)	0.26 (0.13, 0.40)			
RCPM^2^	Before	-0.25 (-0.34, -0.16)	-0.24 (-0.32, -0.15)	27.81 (1,903)	<0.01	0.030
	After	0.14 (0.05, 0.24)	0.12 (0.03, 0.22)			
	Difference	0.39 (0.27, 0.52)	0.36 (0.22, 0.49)			
Performance index^3^	Before	0.13 (0.04, 0.22)	0.12 (0.03, 0.21)	15.49 (1,902)	<0.01	0.017
	After	-0.16 (-0.25, -0.07)	-0.15 (-0.25, -0.05)			
	Difference	-0.29 (-0.42, -0.16)	-0.27 (-0.40, -0.13)			
Total Difficulty score^4^	Before	-0.15 (-0.25, -0.06)	-0.11 (-0.20, -0.02)	0.04 (1,899)	0.847	4.20E-05
	After	-0.05 (-0.15, 0.05)	-0.10 (-0.19, 0.003)			
	Difference	0.10 (0.03, 0.24)	0.01 (-0.12, 0.15)			

*The ANCOVA and mean values were adjusted for child age^1-3^, sex^3,4^, recent illnesses^3^, total difficulties score^1,3^, prosocial behavior score^1,3^, caregivers’ educational status^1,4^, head of the household educational status^1,2^, HOME score^1-4^, household food security^1,2,4^ and wealth index^1-4^. RCPM: Raven’s coloured progressive matrices.

ANCOVA analysis showed that digit span and RCPM were significantly higher whereas performance index of the visual search task was significantly lower in post-COVID-19-lockdown children compared to pre-COVID-19-lockdown children. Performance index for visual search task was lower by 0.27 SD (95%CI: -0.40, -0.13) among post-COVID-19-lockdown compared to pre-COVID-19-lockdown children (**Table 3**). The effect size of the lockdown was small (F(1,900) = 15.49, p < 0.01, η^2^_p_ = 0.017). Children, post-COVID-19-lockdown, scored higher by 0.26 SD (95%CI: 0.13, 0.40) of digit span and 0.36 SD (95%CI: 0.22, 0.49) of RCPM compared to children pre-COVID-19 (**Table 3**). The effect sizes of the lockdown were small for both digit span (F(1,902) = 14.21, p < 0.01, η^2^_p_ = 0.016) and RCPM (F(1,903) = 27.81, p < 0.01, η^2^_p_ = 0.030).

### Change in cognitive and behavioral outcomes–Subgroup analysis

**Fig 1:**
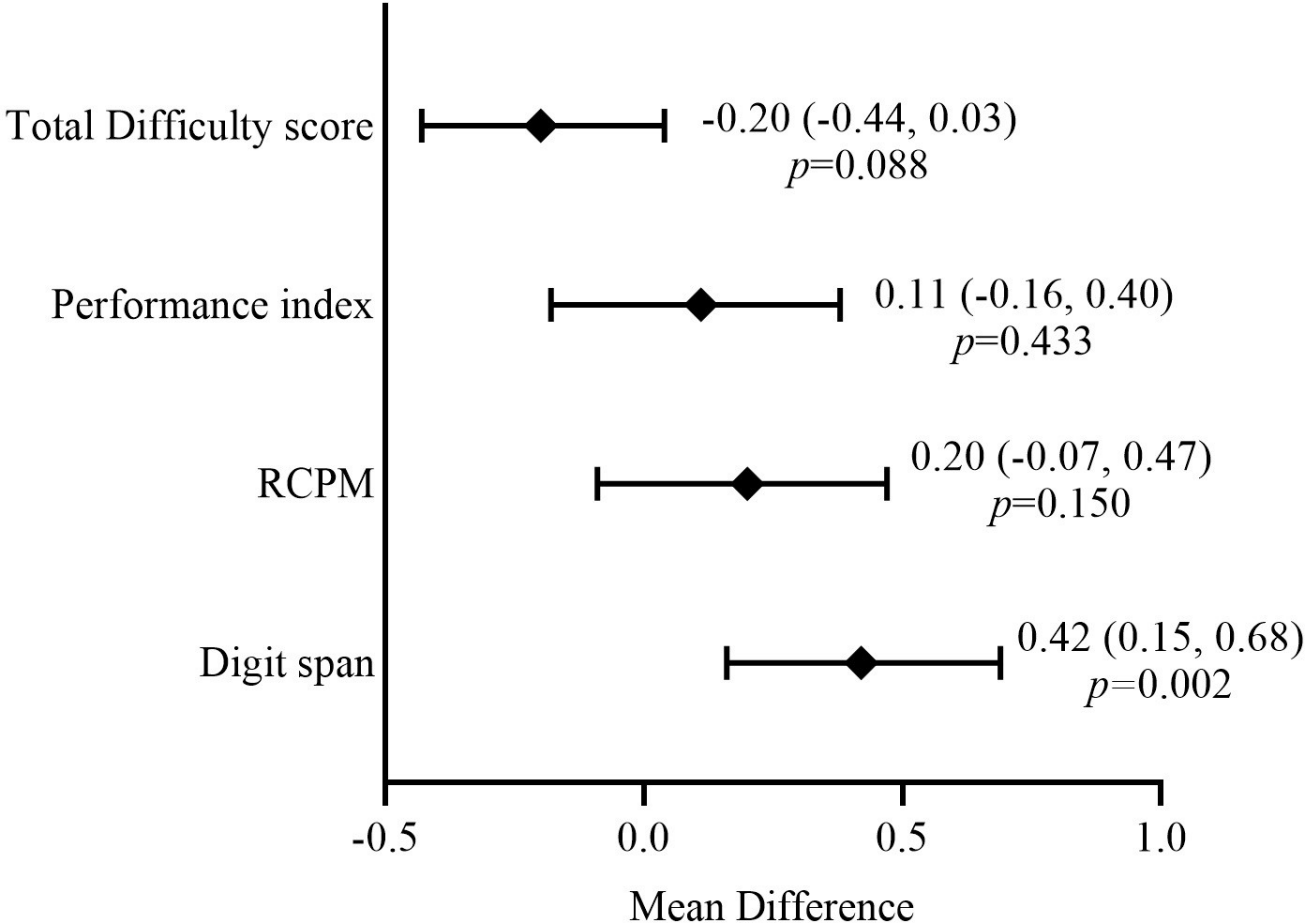
Pre- and post-COVID-19-lockdown mean differences of cognitive developmental and behavioral difficulty of schoolchildren at Arba Minch HDSS (n = 88). [*Adjusted values for child age, sex, recent illnesses, total difficulties score (not included for total difficulty score), prosocial behavior score, caregivers and head of the household educational status, HOME score, household food security and wealth score. RCPM: Raven’s coloured progressive matrices].

The subgroup analysis conducted among children who participated in both surveys showed that the mean difference of the child’s digit span increased by 0.42 SD (95%CI: 0.15, 0.68) post-COVID-19 lockdown. The decrease in behavioral difficulty score post-COVID-19 lockdown was marginal (p = 0.088). However, RCPM and performance index for visual search task did not show significant difference between pre- and post-COVID-19 lockdown among children who participated in both surveys (Fig 1).

### Modifying effects of behavioral difficulties and home environment on the associations between cognition and pre- and post-COVID-19-lockdown

There was a significant interaction between the survey period and prosocial behaviors of the children for the RCPM and performance index. RCPM was higher among children with more behavioral difficulties compared to children with no difficulties (mean difference between post- and pre-COVID-19-lockdown of 0.59 SD (95%CI: 0.31, 0.87) compared to 0.29 SD (95%CI: 0.14, 0.44), respectively). Similarly, RCPM was higher among children with lower prosocial behavior score compared to children with normal prosocial behavior score (mean difference between post- and pre-COVID-19-lockdown of 0.83 SD (95%CI: 0.55, 1.11) compared to 0.24 SD (95%CI: 0.09, 0.38), respectively). The difference in mean between post- and pre-COVID-19-lockdown on performance index of visual search task was lower among children with no behavioral difficulties (-0.33 SD; 95%CI: -0.48, -0.18) and children with normal prosocial behavior (-0.35 SD; 95%CI: -0.50, -0.20). However, there was no significant difference among children with behavioral difficulties and who had lower prosocial behavior during the two surveys. During the post-COVID-19-lockdown, children from under stimulating home environments had higher behavioral difficulties by 0.37 SD (95%CI: 0.11, 0.63) compared to those participated pre-COVID-19-lockdown. However, there was no significant difference among children with no behavioral difficulties during the two surveys (**Table 4**).

**Table 4:** Subgroup analysis of the cognitive developmental and behavioral difficulty of schoolchildren at Arba Minch HDSS before and after COVID-19 lockdown.

Outcome and Subgroup factor	Pre-lockdown		Post-lockdown		Unadjusted difference		Adjusted difference*	
	Mean ± SD	n	Mean ± SD	n	(95% CI)	P	(95% CI)	P
**Digit span** ^**1**^								
HOME score						0.808		0.872
Normal	-0.24 (1.06)	388	0.03 (0.99)	291				
Under stimulated	-0.35 (1.02)	99	-0.04 (1.02)	128				
Total difficulties						0.383		0.376
Normal	-0.24 (1.05)	391	0.07 (0.98)	319				
More difficulties	-0.34 (1.05)	96	-0.17 (1.04)	100				
Prosocial behavior					0.702		0.637
Normal	-0.23 (1.06)	390	0.03 (0.99)	328				
Lower	-0.40 (1.08)	97	-0.06 (1.04)	91				
**Raven’s coloured progressive matrices** ^**2**^								
HOME score						0.319		0.230
Normal	-0.23 (1.04)	388	0.13 (1.00)	291				
Under stimulated	-0.33 (0.86)	99	0.18 (0.99)	128				
Total difficulties						0.096		0.064
Normal	-0.22 (1.00)	391	0.12 (0.98)	319	0.34 (0.19, 0.48)	<0.01	0.29 (0.14, 0.44)	<0.01
More difficulties	-0.39 (1.02)	96	0.22 (1.04)	100	0.60 (0.33, 0.88)	<0.01	0.59 (0.31, 0.87)	<0.01
Prosocial behavior					<0.01		<0.01
Normal	-0.20 (1.00)	390	0.07 (1.01)	328	0.27 (0.13, 0.42)	<0.01	0.24 (0.09, 0.38)	0.002
Lower	-0.45 (1.02)	97	0.40 (0.92)	91	0.85 (0.57, 1.14)	<0.01	0.83 (0.55, 1.11)	<0.01
**Performance index of visual search task** ^**3**^								
HOME score						0.695		0.529
Normal	0.17 (1.04)	388	-0.11 (0.94)	291				
Under stimulated	-0.04 (0.97)	99	-0.29 (1.01)	128				
Total Difficulties					0.117		0.076
Normal	0.17 (1.03)	391	-0.19 (0.99)	319	-0.34 (-0.49, -0.19)	<0.01	-0.33 (-0.48, -0.18)	<0.01
More difficulties	-0.02 (1.02)	96	-0.11 (0.88)	100	-0.09 (-0.37, 0.19)	0.519	-0.05 (-0.33, 0.22)	0.717
Prosocial behavior					0.052		0.018
Normal	0.18 (1.05)	390	-0.18 (0.98)	328	-0.35 (-0.50, -0.21)	<0.01	-0.35 (-0.50, -0.20)	<0.01
Lower	-0.08 (0.91)	97	-0.12 (0.93)	91	-0.04 (-0.32, 0.24)	0.790	0.03 (-0.25, 0.32)	0.821
**Total difficulties score** ^**4**^								
HOME score						0.007		0.002
Normal	-0.28 (1.15)	388	-0.28 (0.99)	291	-0.06 (-0.21, 0.10)	0.449	-0.11 (-0.27, 0.04)	0.154
Under stimulated	0.12 (0.84)	99	0.48 (1.00)	128	0.36 (0.10, 0.63)	0.008	0.37 (0.11, 0.63)	0.005

RCPM: Raven’s coloured progressive matrices; *Adjusted values for child age^1-3^, sex^3,4^, recent illnesses^3^, total difficulties score^1,3^, prosocial behavior score^1,3^, caregivers’ educational status^1,4^, head of the household educational status^1,2^, HOME score^1-4^, household food security^1,2,4^ and wealth score^1-4^. RCPM: Raven’s coloured progressive matrices.

## Discussion

Schooling provides tremendous opportunity for children in many ways, including providing educational materials, interaction with teachers and their age-mates, promoting health-related practices such as personal hygiene, recommended dietary practices, physical activity, and good sleeping habits [30, 31]. During the lockdown period, children were confined at home where their growth, development and social interactions depended to a large extent on their parents. This might have been an opportunity for some households to enhance the interaction between parents and their children, through the involvement of the latter in the family activities, and increased communication and learning skills from their parents. In addition, with the right parenting approaches, family bonds can be strengthened, and psychological need could be met [31, 32]. However, the lockdown measures were more extreme than enhancing the time spent within the family, and have put most of the households at strain [33, 34]. Educational outcomes of the students were negatively affected by school closure, even in settings where options of distance learning were available and offered [35–37].

Our study showed that post-COVID-19-lockdown children had higher working memory and non-verbal intelligence, but lower attention and concentration compared to their peers who were assessed prior to the COVID-19-lockdown. However, children’s behavioral difficulties did not differ between pre- and post-COVID-19-lockdown. Children who participated in both surveys showed an improvement in working memory during post-COVID-19-lockdown, with no difference in behavioral difficulties, thus confirming the cross-sectional findings.

Similar findings were reported among undergraduate psychology students in Spain, who were found to have an improved working memory after long-term home confinement [38]. A study that explored the subjective cognitive functioning and their possible interplay related to COVID-19-lockdown among adult Italians found that their memory ability, which was evaluated by prospective and retrospective memory questionnaire, has improved [39]. Different results were found among adults from North America and British Isles. This study reported that participants who had high COVID-19-related anxiety tend to have poorer n-black performance [40], one of the tools used to assess working memory performance [41, 42]. While the other tools used for assessing working memory, which included running memory paradigm, the span paradigm or the selective updating paradigm, indicated that COVID-19 related anxiety did not affect working memory performance [40]. The research supports that working memory skill of children grows more during school-year months compared to its growth during the summer holidays [43]. It suggests that school environment provides a unique opportunity for child’s growth, learning and development, despite the difference in the impact between the time spent outside school because of school vacation time and the home confinement measures of COVID-19-lockdown [43, 44].

Our findings indicated that non-verbal intelligence of schoolchildren was higher post-COVID-19-lockdown. Findings from a study conducted on Norwegian schoolchildren explained that the extra-ordinary home confinement situation might have provided the child with freedom to engage in self-entertaining, and in manipulating objects and observing their interrelations. It reported that isolated children perform better in the logical operations on objects than their counterparts [45]. This could explain why children post-COVID-lockdown scored higher, especially in non-verbal intelligence, which is not affected significantly by verbal stimulation. Our finding also showed that the mean difference in non-verbal intelligence between post- and pre-COVID-19-lockdown restriction were higher among lower prosocial behavior score and among children with more behavioral difficulties compared to children with normal prosocial behavior score or normal difficulties score. The limitation of social interaction among children post-COVID-19-lockdown may have impacted the abilities of children with normal prosocial behaviors compared to those with lower prosocial behaviors. Similarly, this situation may have disadvantaged children with lower behavioral difficulties.

Although the effect was small, our study showed that schoolchildren who participated before the COVID-19 pandemic had higher attention levels compared to those who participated after the COVID-lockdown. Our findings are in line with the body of evidence from high-income countries. A study among Italian adults found that participants performed significantly lower in attention and concentration after the lockdown [39]. A study on children from Italy and France showed that children manifested worse emotional status after the lockdown, probably due to the limited interaction in person [35]. A report based on US adolescents indicated poor mental health and increased stress, assessed using prolonged screen use [46]. Worse emotional status, stress and change in sleep behavior [47] might be related to a decrease in attention and concentration of schoolchildren. On top of the disease (COVID-19) related effect on the attention among those who were infected [48], the lower attention and concentration of the children post-lockdown might be related to high negative and low positive emotions that was experienced by the public [49]. The study conducted among young adults in Spain showed that there was no significant difference between pre-pandemic and during-confinement in the score of attention capacity, which was assessed by change location task [38]. We also found that attention and concentration of the children decreased post COVID-19-lockdown, among children with no behavioral difficulties and normal prosocial behaviors.

This report showed that there was no difference in behavioral difficulties among schoolchildren participating in pre- and post-COVID-19-lockdown restrictions. In addition, the prevalence of children with abnormal behaviors did not show difference between the two periods of data collection. Similarly, a longitudinal analysis based on the pre- and during COVID-19 lockdown in England indicated that there was no significant difference in SDQ-based mental health assessment of the schoolchildren [50]. Another UK-based longitudinal study which collected data since the beginning of the lockdown indicated that the symptoms of mental health problem increased during the beginning of lockdown, stabilized during the restriction eased, and decreased when children returned to school [51]. This might explain the non-differences between the scores of children pre- and post-COVID-19-lockdown found in our study, as the post-COVID-19-lockdwan data was collected after the children were back to the school. Although there was no difference in behavioral difficulties among children participating in pre- and post-COVID-19-lockdown, we found that children from under stimulating home environment and post-COVID-19-lockdown had higher behavioral difficulties compared to pre-COVID-19-lockdown. The restriction posed by the pandemic had impacted the children to use of the home environment as the only or primary physical and social environment. Although under stimulating home environment had been linked to behavioral problems under normal circumstances (without restrictions related to the pandemic). A study from the US supported the relationship between the home environment and behavioral problems among children. It indicated that those from more positive HOME score households had fewer behavioral problems [52].

Only three cognitive domains were included in this analysis (non-verbal intelligence, working memory and attention). We used a cross-sectional design, which is used to explore a cumulative change. However, the design does not assess the change at the individual level. We have conducted the subgroup analysis among 88 children who participated in both surveys, confirming the cross-sectional findings concerning the children’s working memory and behavioral difficulty. There is a shortage of literature to compare our findings specifically concerning the impact of COVID-19 lockdown on cognition of schoolchildren in low income countries. As an example, at the country level, the number of enrolled children decreased by approximately 2.1 million after the COVID-19 lockdown compared to the pre-COVID-19 lockdown period [53, 54], highlighting the significant impact of the lockdown. However, it is important to note that this aspect was not captured in our study.

## Conclusions

The attention and concentration domain of the schoolchildren post-COVID-19-lockdown was lower compared to pre-COVID-19-lockdown with small effect size. On the contrary, schoolchildren had improved working memory and non-verbal intelligence post-COVID-19 lockdown, with strong evidence supporting the improvement of working memory. In addition, behavioral difficulties due to COVID-19 lockdown were not confirmed. Factors that were at play in improved working memory and non-intelligence of the schoolchildren while away from the school environment should be explored and used for enhancing the child development. Research should also be carried out to find solutions and interventions that can support schoolchildren and reduce the impact of similar situations in the future.
